# Alcohol use effects on adolescent brain development revealed by simultaneously removing confounding factors, identifying morphometric patterns, and classifying individuals

**DOI:** 10.1038/s41598-018-26627-7

**Published:** 2018-05-29

**Authors:** Sang Hyun Park, Yong Zhang, Dongjin Kwon, Qingyu Zhao, Natalie M. Zahr, Adolf Pfefferbaum, Edith V. Sullivan, Kilian M. Pohl

**Affiliations:** 1 Department of Robotics Engineering, Daegu Gyeongbuk Institute of Science and Technology, Daegu, South Korea; 2 Colin Artificial Intelligence Lab, Richmond, BC Canada; 3 Department of Psychiatry & Behavioral Sciences, Stanford University, Stanford, CA 94305 USA; 4 Center for Health Sciences, SRI International, Menlo Park, CA 94025 USA

## Abstract

Group analysis of brain magnetic resonance imaging (MRI) metrics frequently employs generalized additive models (GAM) to remove contributions of confounding factors before identifying cohort specific characteristics. For example, the National Consortium on Alcohol and NeuroDevelopment in Adolescence (NCANDA) used such an approach to identify effects of alcohol misuse on the developing brain. Here, we hypothesized that considering confounding factors before group analysis removes information relevant for distinguishing adolescents with drinking history from those without. To test this hypothesis, we introduce a machine-learning model that identifies cohort-specific, neuromorphometric patterns by simultaneously training a GAM and generic classifier on macrostructural MRI and microstructural diffusion tensor imaging (DTI) metrics and compare it to more traditional group analysis and machine-learning approaches. Using a baseline NCANDA MR dataset (N = 705), the proposed machine learning approach identified a pattern of eight brain regions unique to adolescents who misuse alcohol. Classifying high-drinking adolescents was more accurate with that pattern than using regions identified with alternative approaches. The findings of the joint model approach thus were (1) impartial to confounding factors; (2) relevant to drinking behaviors; and (3) in concurrence with the alcohol literature.

## Introduction

After birth, the human brain undergoes profound change that continues throughout adolescence and into young adulthood^1^. A consensus of cross-sectional and longitudinal magnetic resonance imaging (MRI) studies suggests that cortical gray matter volume declines and the cortical mantle thins^2,3^, but white matter volume, microstructural organization, and myelination of fiber tracts increase^4,5^, during healthy adolescent development. In this developmentally critical second decade of life, young people commonly engage in risky behaviors, including consumption of alcohol. A recent U.S. survey estimates that 66% of 18-year-olds have drunk alcohol and about 25% report getting drunk^6^. A rising incidence of binge drinking may put developing youth at particularly high risk for deviations from the normal trajectory of brain development^7^. Longitudinal studies of heavy relative to minimal drinking during adolescence report acceleration of gray matter volume shrinkage, attenuation of white matter growth^8^, and decreased fiber integrity^9^. Similar but subtler developmental changes have been detected in youth who drink regularly, if not heavily^10^. Despite such reports of quantifiable effects of drinking on normal neurodevelopmental trajectories, weak effects may be difficult to extricate using traditional, hypothesis-driven methods^11^ and may be enhanced by the use of machine-learning approaches to determine group separating characteristics^12^.

In neuroimaging studies, identifying group differences using classification approaches can be straight forward if the groups are of equal sample size and matched with respect to demographic factors such as age, sex, and ethnicity^13–15^. However, a challenge of many neuroimaging studies is statistical power, particularly given the number of potentially confounding factors^16^. For example, the National Consortium on Alcohol and Neurodevelopment in Adolescence (NCANDA)^17^, a landmark longitudinal study supported by the National Institute on Alcohol Abuse and Alcoholism and the National Institutes of Health Big Data to Knowledge initiative, has been collecting MRI and neuropsychological data in adolescents and young adults to (1) expand knowledge about normal brain maturation; (2) document changes following initiation of moderate-to-heavy alcohol consumption; and (3) identify imaging markers that predict early-onset alcohol use disorder (AUD). The number of youth with a notable history of alcohol consumption at baseline was small^17^. To power this investigation adequately, however, the study also recruited youth with minimal alcohol exposure at baseline that had a high risk for transitioning to the AUD phenotype during the course of the 10-year study.

One popular approach for analyzing unbalanced data sets is to include only subsets of the collected sample matched with respect to basic demographic variables. For example (in support of the first aim of the NCANDA study), age-matched samples selected from another large cohort study, the ‘Pediatric Imaging, Neurocognition, and Genetics’ data set confirmed the longitudinal brain developmental patterns identified in the minimal drinking adolescents of the NCANDA cohort^3^. Specific to the NCANDA cohort and its second aim, the study also reported smaller and thinner frontal and temporal cortices for the group initiating moderate-to-heavy alcohol consumption relative to the minimally-drinking group. Matching cohorts, however, is not always successful in revealing significant group differences. For example, analyses of diffusion tensor image (DTI) data from demographically-matched subsets of the NCANDA study did not reveal effects of moderate-to-high drinking on DTI metrics (*i*.*e*., regional fractional anisotropy, mean diffusivity, axial diffusivity, and radial diffusivity)^4^. This was surprising given evidence that excessive alcohol consumption in adults disrupts white matter microstructure of select fiber systems^18–21^.

An alternative approach to analyzing unbalanced data is to include the entire sample, but to remove the effects of confounding variables before performing group analysis^12,22–27^. Regression approaches, such as the ‘ordinary’ generalized additive model (GAM), remove the effects of confounding factors by first modeling the relationship between the dependent variable (*e.g*., volume of the corpus callosum) and confounding factors (*e.g*., age) on a subset of the sample (*e.g*., minimal alcohol-consuming healthy controls)^3^, then using that model to remove the effect of confounding factors from each dependent variable so that residuals of the raw metric are used in group analyses. However, GAM often suffers from sensitivity to noise, as demonstrated, for example, by the variance in age associated with peak white matter microstructural maturation^4^. Robust regression claims to address the sensitivity issue by separately modeling the effects of confound and noise in MRI metrics^28^. While robust regression has often been used in large neuroimaging studies^29^, the noise model requires a-priori specification, which can reduce the power of the analysis. For example, a cautious threshold for accounting for noise generally results in a robust GAM but the effects of confounding variables are then estimated on a notably reduced sample size. A small sample generally fails to capture comprehensive effects of confounding factors and the resulting GAM is thus likely inaccurate. We hypothesized that typical sequential use of the GAM to isolate the effects of confounding variables on MR metrics would minimize information relevant for distinguishing groups (*e.g*., adolescents with a drinking history relative to those without a significant drinking history).

**Figure 1:**
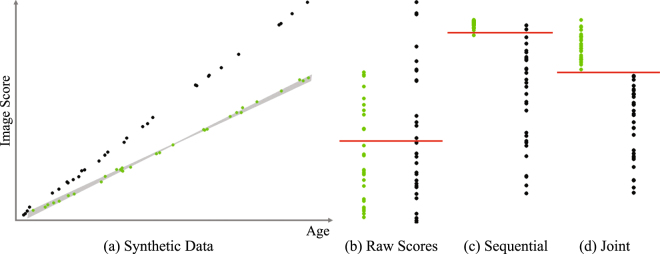
Synthetic example. (**a**) Raw image scores of minimal drinkers (green) and regular drinkers (black). Each group consisted of 30 samples with ages randomly chosen between 17 and 21 years. The gray region indicates the solution space for the optimal regression line defining the GAM mode. (**b**) Classification based on raw scores did not accurately distinguish the two groups (red line). (**c**) Removing age-effects from the raw scores via GAM clearly separated both groups with only a few mislabeled sample points, and (**d**) classification using the joint method resulted in perfect group separation.

To test the hypothesis, we apply a machine learning approach to the baseline NCANDA neuroimaging data set. Our proposed approach, referred to as Joi-GAM-Class (for joint GAM classification) simultaneously determines optimal parameters (1) of a GAM (for removing the influence of confounding factors) and (2) a logistic classifier (for cohort classification). The classifier identifies a subset of variables (*i*.*e*., residual scores of imaging metrics) that is most informative for differentiating minimal from regular drinking youth. We refer to this subset of brain measurements as pattern. To identify a pattern, the classifier’s search for informative brain metrics is constrained to subsets of a certain size, enforced by embedding ‘sparsity constraints’ into the classification model^12^. To help with an initial understanding of the method used herein, Fig. 1 presents the output of three approaches analyzing a synthetic data set. Figure 1(a) plots an arbitrary image metric (y-axis) relative to age (x-axis). The green dots represent the imaging metrics of the minimal drinkers and the black ones of the regular drinkers. For both cohorts, the metric is clearly effected by age, a confounding factor also in the NCANDA data. The effects of age outweigh the effects of group when the classifier is applied directly to raw imaging metrics (*i*.*e*., not residuals) as the two cohorts are not separated accurately (Fig. 1(b)). Figure 1(c) shows a few samples that are mislabeled by classification based on residual scores of raw imaging metrics, *i*.*e*., after the confounding effects of age are removed via robust GAM. The GAM was parameterized based on the imaging metrics of the control group, *i*.*e*., the minimal drinkers. As is true with real data, however, the noise associated with raw imaging metrics made it highly unlikely to recover the ‘true’ age effect. Instead the data allow for a variety of plausible solutions shown schematically in the gray region outlined in Fig. 1(a). Within this set of possible solutions, the robust regression chose the solution that best fits *a-priori* assumptions. The assumptions were defined through specific settings of the underlying optimization algorithm, which were not specific to the classification task. By contrast, our joint optimization approach selected the GAM model so that the classifier perfectly separated the two cohorts (Fig. 1(d)).

To complete hypothesis testing, we cross-validate our joint algorithm approach (*i*.*e*., Joi-GAM-Class) against alternative implementations using the baseline NCANDA imaging data set. The data set consists of structural MRI and microstructural DTI metrics collected in 705 adolescents: 671 that are minimal (no-to-low) drinking and 34 that are regular drinkers. The GAM is defined with respect to age and socioeconomic status, because these two variables are not matched across the two cohorts (and are therefore confounding variables). To apply cross-validation, a popular method to measure accuracy of machine learning approaches, the total data set is divided into subsets (*i*.*e*., folds) in which the cohorts in each subset are matched with respect to demographic factors other than age and socioeconomic status. Each implementation uses one subset for training. The accuracy of patterns identified during training is then evaluated on the second subset to ensure that solutions are not specific to the first subset. This process is repeated with the second subset used for training and first for testing. The test accuracy of classifiers is summarized with accuracy scores, which include measures for testing the resistance of implementations to confounding factors. Furthermore, we compute *p*-values representing the statistical significance of accuracy scores and patterns identified by each implementation. Here, we are the first to report progress on the third aim of NCANDA (*i*.*e*., identify imaging markers that predict early-onset AUD) by presenting patterns of neuromaturation that are impartial to confounding effects (such as age) and correctly classify adolescents who drink regularly.

In a conference paper^30^, we first discussed the idea of jointly parameterizing GAM and classification to analyze two independently collected structural MRI data sets of participants ranging in age from 60 to 72 years (*N* = 74). The first data set contained participants infected with the Human Immunodeficiency Virus (HIV) and effected by HIV-Associated Neurocognitive Disorder as well as demographically matched controls. The second data set, which was matched to the first one, contained individuals diagnosed with Mild Cognitive Impairment and a control cohort. In the conference submission, the GAM was used to remove the effect of acquisition differences between the two data sets and the classifier to identify differences between HIV-Associated Neurocognitive Disorder and Mild Cognitive Impairment. The experiment revealed that our joint approach is more accurate than sequential methods in identifying group differences based on data not ideally constructed for classification. Here, we confirm this finding on the NCANDA data set.

## Results

### Comparison of Sequential and Joint Approaches

Our experiments on the NCANDA data set revealed that our joint approach Joi-GAM-Class (based on MRI and DTI metrics) was indifferent to confounding factors (*i*.*e*., age and socioeconomic status) and more accurate than alternative implementations, listed here:

- **No-GAM-Class**: performed sparsity constrained classification on raw image scores (*i*.*e*., omitting GAM); the benchmark for analysis without removing the effects of confounding factors.
- **Seq-GAM-Class**: popular sequential approach first parameterized an ordinary GAM and then performed sparsity-constrained classification.
- **Seq-GAM**_***Rob***_-**Class**: sequentially executed robust regression and sparse classification; the alternative to Seq-GAM-Class that accounted for image noise.
- **Joi**_***STR***_-**GAM-Class**: the proposed joint model confined to the structural (STR) MRI metrics; the only other implementation indifferent to the confounding factors.
- **Joi**_***DTI***_**-GAM**-**Class**: the proposed joint model confined to microstructural DTI metrics; as with Joi_*STR*_-GAM-Class, this method provided a benchmark for single-image modality analysis.
- **Joi**_***OPT***_-**GAM-Class**: a simplified version of our proposed joint model suitable for optimizing group separation, but not indifferent to the effects of confounding factors.

Note, Table S2 of the supplement lists these and all other acronyms used throughout the article.

We measured the accuracy of each implementation using two-fold cross-validation. After training each implementation on the training data to classify minimal and regular drinkers, we measured their accuracies on the testing data by reporting sensitivity, specificity, Area Under the receiver operating characteristic Curve (AUC), and ‘normalized-accuracy’. ‘Normalized-accuracy’ computed the accuracy of an implementation in correctly labeling samples while accounting for differences in sample size between the two cohorts. To ensure the indifference of an implementation to the effects of confounding variables, we also reported ‘matched-accuracy’. To compute ‘matched-accuracy’, we first defined a subset of the test data in which the cohorts were matched with respect to all known demographic scores including age, socioeconomic status, and cohort size and then re-computed the normalized-accuracy with respect to this subset. We set a threshold for labeling an accuracy score as significant at *p* ≤ 0.002 based on a two-tailed Fisher’s exact test^31^ (*i*.*e*., the probability of a classifier’s output to be generated by randomly assigning samples to cohorts) or the DeLong’s test^32^ *i*.*e*., (the probability of the output of one implementation to be generated by another implementation). This significant threshold was considered conservative as the number of implementations compared herein was small^4^. Unless otherwise stated, significant findings refer to the outcome of the Fisher’s exact test.

Desirable implementations were those with significant normalized-accuracy and significant matched-accuracy. For each implementation, indifference to the effects of the confounding variable ‘age’ was calculated using the two-tailed Fisher’s exact test to measure the ability of the relevant classifier to cleanly separate minimal (no-to-low) alcohol-exposed adolescents into an older (*i*.*e*., above the age of 15.4; *N* = 335) and younger cohort (*i*.*e*., below the age of 15.5; *N* = 336). The two cohorts were matched with respect to all demographic factors (*i*.*e*., socioeconomic status, supratentorial volume, sex, ethnicity, scanner) except age. Implementations with p > 0.01 passed the age-test as the effect of age was non-existent or magnitudes weaker than the effects of regular drinking. Thus, desirable implementations that also passed the age-test were considered informative with respect to distinguishing regular drinkers from minimal alcohol exposed adolescents. Critically, all implementations passed the socioeconomic status test, *i*.*e*., a replication of the age-test applied to this variable. We thus omit discussion of this test.

Table 1 summarizes results. The classifier without data harmonization (No-GAM-Class) was the only implementation, whose ‘normalized-accuracy’ score was significantly lower than chance. The sequential implementations (Seq-GAM-Class and Seq-GAM_*Rob*_-Class) had significant ‘normalized-accuracy’ scores but non-significant ‘matched-accuracy’ scores. Compared to those implementations, the joint methods reported higher AUC, normalized-accuracy, and matched accuracy scores. Although specificity was higher than sensitivity for all implementations, the difference between these was substantially smaller for the joint approaches. The smallest difference was observed for Joi_*STR*_-GAM-Class (sensitivity: 70.6%; specificity: 76.9%). Joi_*STR*_-GAM-Class was also informative as it passed the age-test and had significant normalized-accuracy and matched-accuracy scores. Among the joint approaches, the accuracy score was diminished when only DTI metrics were used (*i*.*e*., Joi_*DTI*_-GAM-Class): this implementation also failed the age-test and did not have a significant matched-accuracy score. Joi_*Opt*_-GAM-Class failed the age-test and had the largest difference between normalized-accuracy and matched-accuracy scores (dropped by 12.6%), but it achieved the highest accuracy score (80.8%). Joi-GAM-Class passed the age-test, had a high accuracy score, and the smallest difference between normalized-accuracy (75.9%) and matched-accuracy (77.1%) scores. These accuracy scores were higher than those of the only other informative implementation (*i*.*e*., Joi_*STR*_-GAM-Class). Joi-GAM-Class was also the only implementation that was significantly better than No-GAM-Class and Seq-GAM-Class. On a trend level (p < 0.0003), it was also better than Seq-GAM_*Rob*_-Class.

**Table 1:** Sensitivity, specificity, Area Under the receiver operating characteristic Curve (AUC), normalized-accuracy, matched-accuracy, and age-test (testing for the effect of age) of each implementation.

Method	Sensitivity (%)	Specificity (%)	AUC (%)	Normalized-Accuracy	Matched-Accuracy	Age-Test p-value
No-GAM-Class	20.6	94.6	74.7	57.4^*^	57.4^*^	0.0001
Seq-GAM-Class	32.4	94.0	71.8	**62.9** ^*^	58.8^*^	0.0005
Seq-GAM_*Rob*_-Class	32.4	94.0	78.1	**63.2** ^**+**^	60.3^+^	0.0002
Joi_*STR*_-GAM-Class	70.6	76.9	81.3	**73.7**	**72.1**	**0.0222**
Joi_*DTI*_-GAM-Class	61.8	83.2	78.8	**72.5**	67.7	<0.0001
Joi_*OPT*_-GAM-Class	70.6	91.1	83.8	**80.8**	66.2	<0.0001
Joi-GAM-Class	67.6	84.2	83.2	**75.9**	**76.5**	**0.2057**

Marked in bold were favorable accuracy scores (significant ones with *p* ≤ 0.002) and age-tests (p-values > 0.01). Of all implementations, only the joint methods Joi_*STR*_-GAM-Class and Joi-GAM-Class were indifferent to age. Of those two, Joi-GAM-Class reported the higher accuracy scores. It was also the only method, whose matched-accuracy score was higher than the normalized one. ‘*’marks significantly worse scores (*p* ≤ 0.002) and ‘^+^’ marks scores trending to being significantly worse (p < 0.003) than Joi-GAM-Class.

### Pattern Analysis

**Figure 2:**
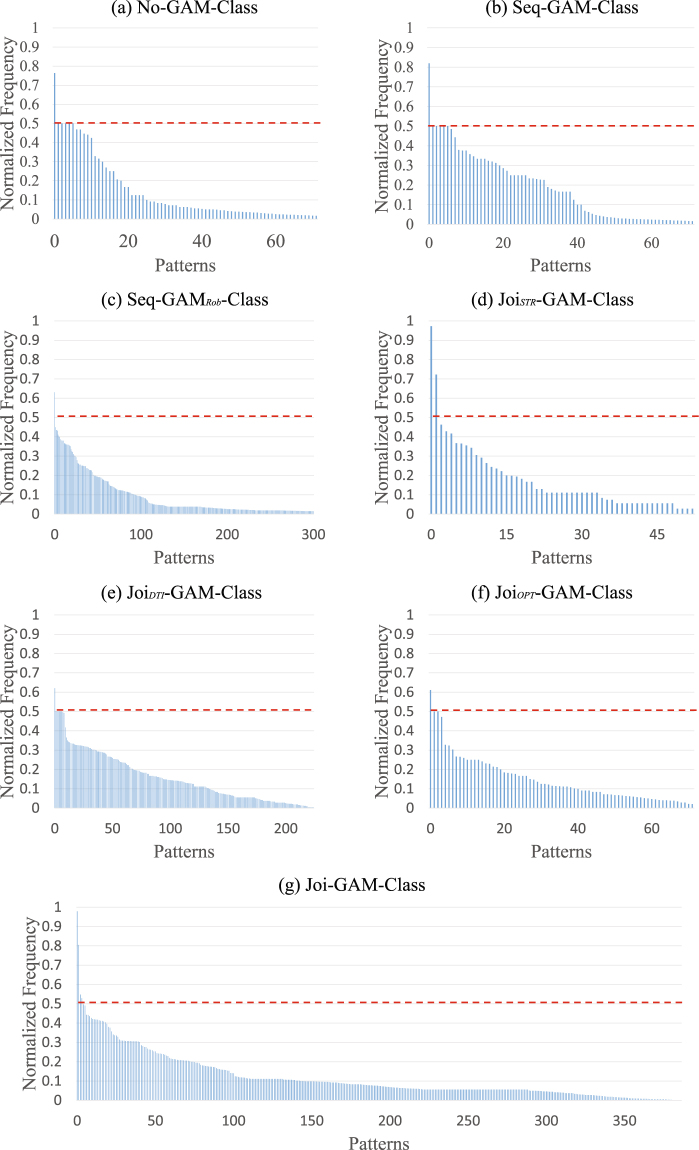
Normalized frequency of patterns selected by each implementation (across all training runs). Unique patterns identified by each approach (x-axis), sorted in descending order according to their normalized frequency (y-axis). A pattern is a set of regional metrics that our method deemed informative for distinguishing regular drinking adolescents from minimal ones. Patterns were labeled as ‘dominant’ if their frequency was above a ‘red dashed line’ threshold (*i.e*., they appeared in more than 50% of suitable runs). While the curve defined by the normalized frequency across all patterns quickly dropped off for all implementations, Joi-GAM-Class had the highest number of dominant patterns. These results indicate that Joi-GAM-Class identified informative patterns across runs more consistently than any other implementation.

As part of cross-validating an implementation, training consisted of parameter exploration, *i*.*e*., recording the identified pattern and corresponding accuracy for different parameter settings of the implementation. A pattern consists of a small number of MR-derived metrics that the implementation deemed informative for distinguishing the two cohorts. Figure 2 plots the normalized frequency of unique patterns identified by each implementation across all training runs. The following lists each implementation by the number of unique regions identified: Joi_*STR*_-GAM-Class (53 patterns), No-GAM-Class (72 patterns), Seq-GAM-Class (72 patterns), Joi_*OPT*_-GAM-Class (72 patterns), Joi_*DTI*_-GAM-Class (225 patterns), Joi-GAM-Class (381 patterns), and Seq-GAM_*Rob*_-Class (853 patterns). Interestingly, Joi-GAM-Class recorded four informative (and dominant) patterns each appearing in at least 50% of the training runs.

**Figure 3:**
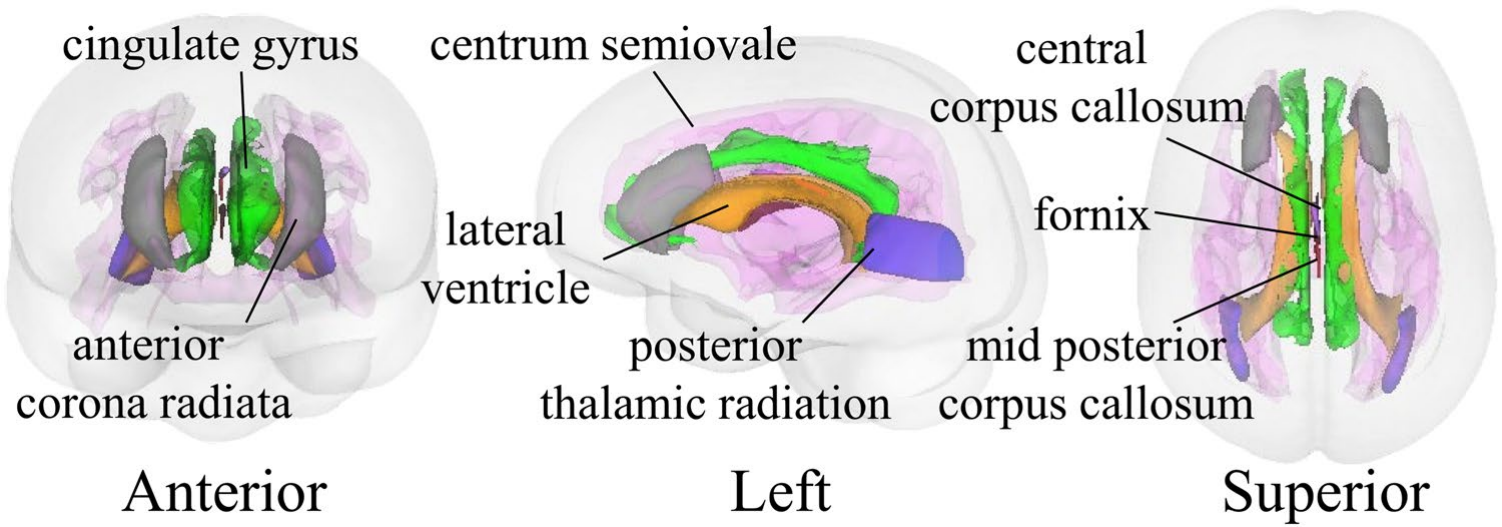
3D models of the eight brain regions selected by the four most frequent appearing patterns of Joi-GAM-Class. The boundaries of the lateral ventricle, mid posterior corpus callosum, and central corpus callosum were defined according to the SRI24 atlas and the centrum semiovale according to the Desikan-Killiany atlas. The regions of the diffusion-weighted measures (i.e., the anterior corona radiate, posterior thalamic radiation, and fornix) were defined according to the Johns Hopkins University atlas. Both Desikan-Killiany atlas and Johns Hopkins University were non-rigidly aligned to the SRI24 atlas to generate these 3D models of the select regions via 3D Slicer.

The four informative patterns of Joi-GAM-Class consist of the MR metrics listed in Table 2. The most frequently selected pattern (97.8%) consisted of the volumes of lateral ventricles and mid posterior corpus callosum. The second pattern (80.5%) included the first two MR metrics and two additional structural MRI metrics (*i*.*e*., volumes of centrum semiovale and central corpus callosum). The third pattern (54.7%) added DTI metrics fractional anisotropy of anterior corona radiata and posterior thalamic radiation) and the fourth (52.9%) included also axial diffusivity of fornix and volume of cingulate gyrus (Fig. 3). Thus, this implementation provided consistency in the identified patterns.

**Table 2:** Informative patterns of Joi-GAM-Class and corresponding selected regions.

Regions	Measurement Type	Patterns				Frequency (%)	Normalized-Accuracy	Matched-Accuracy
		1	2	3	4			
Lateral ventricle	Volume	X	X	X	X	99.0	60.1	66.2
Mid posterior corpus callosum	Volume	X	X	X	X	97.9	63.0	63.2
Centrum semiovale	Volume		X	X	X	99.2	61.1	55.9
Central corpus callosum	Volume		X	X	X	79.3	64.0	64.7
Anterior corona radiata	Fractional anisotropy			X	X	96.3	**80.0**	67.6
Posterior thalamic radiation	Fractional anisotropy			X	X	58.0	**75.4**	66.2
Fornix	Axial diffusivity				X	90.0	**67.1**	66.2
Cingulate gyrus	Volume				X	61.4	53.0	51.5
Frequency (%)		97.8	80.4	54.7	52.9			
Normalized-Accuracy		**65.0**	**64.8**	**74.6**	**79.4**			
Matched-Accuracy		69.1	66.2	**70.6**	**79.4**			

Accuracy scores in bold were significantly different from chance (*p* ≤ 0.002).

Alternative implementations also frequently selected the previously mentioned regions. The only MRI metrics not used by Joi-GAM-Class were the mean diffusivity of the corticospinal tract selected by Seq-GAM-Class, and the axial diffusivity of the medial lemniscus selected by Seq-GAM_*Rob*_-Class.

Table 2 also lists the normalized- and matched-accuracy scores for the logistic classifier confined to the residual scores of the four patterns selected by Joi-GAM-Class. The fourth pattern, which included the MRI metrics of the other three patterns, had equivalent normalized-accuracy and matched-accuracy scores (79.4%). The classifier based solely on a single MRI metric achieved accuracy scores below 70% for most regions. The classifier based on the fractional anisotropy of the anterior corona radiata (normalized-accuracy: 80%) and the posterior thalamic radiation (normalized-accuracy: 75.4%) were exceptions, but their matched-accuracy scores were below 70%.

**Figure 4:**
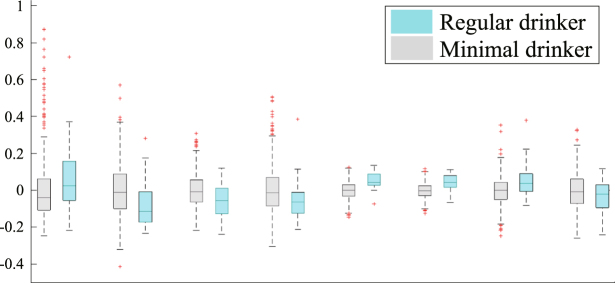
Box plots of the residual scores of the brain regions selected by the informative patterns of Joi-GAM-Class. The central line in the box is the median, the two edges are the 25^th^ and 75^th^ percentiles, the whiskers extend to one-and-a-half times the interquartile range, and red pluses are the outliers. Regional scores marked in bold were significantly different between the regular drinking and minimal alcohol exposed group (p ≤ 0.002).

**Figure 5:**
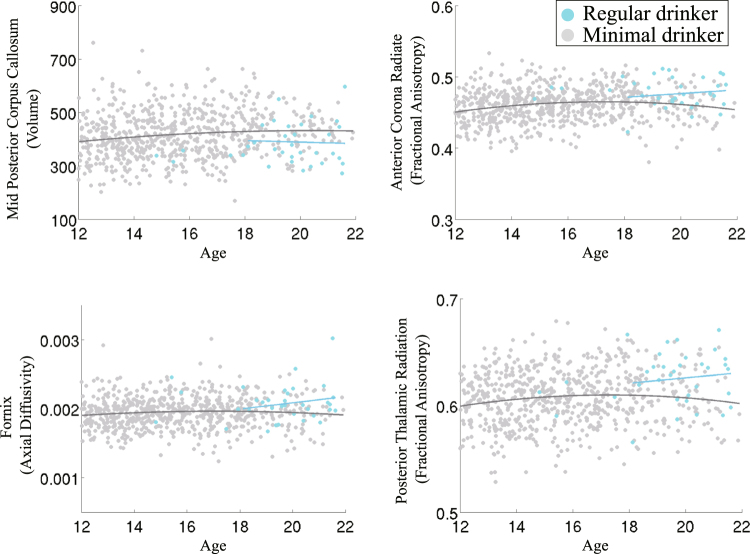
Age-related plots of the residual imaging scores that were significantly different between regular drinkers and minimal alcohol exposed adolescents. The gray regression lines are inferred from the residual imaging scores of the minimal alcohol exposed cohort whereas the blue regression line is that of the regular drinkers age 18 or older. Omitted from the regression are regular drinkers below the age of 18 due to their sparse age-related distribution. Relative to the minimal alcohol exposed cohort, the older regular drinkers show an age-related increase in three diffusion based imaging scores and a slight decrease in the volume of the mid posterior corpus callosum over age.

Regarding group differences (see Figs 4 and 5), the volume of the mid posterior corpus callosum was significantly smaller ($p=0.0002$) in regular drinkers relative to minimal alcohol-drinking adolescents. The axial diffusivity of the fornix ($p=0.0005$) and the fractional anisotropy of the anterior corona radiata ($p < 0.0001$) and posterior thalamic radiation ($p < 0.0001$) were significantly higher in the regular drinking adolescents relative to those with minimal alcohol-exposure.

## Discussion

Joi_*STR*_-GAM-Class and Joi-GAM-Class were the only successful approaches for identifying regular drinking on a subject level. This finding supports our central hypothesis that typical sequential use of the GAM to isolate the effects of confounding variables on MR metrics would minimize information relevant for distinguishing groups (*e.g*., adolescents with a drinking history relative to those without a significant drinking history). Joi-GAM-Class (*i*.*e*., the more accurate of these methods) selected patterns that included structural MRI volumes of the lateral ventricles, centrum semiovale, corpus callosum, and cingulate gyrus and microstructural DTI measures of the fornix, corona radiata, and thalamic radiations. The integrity of each of these regions has been reported to be affected by alcohol misuse in studies using more traditional, hypothesis-driven, morphometric group analysis. When this type of analysis was applied to those eight MRI metrics, only four of them showed significant group differences on the NCANDA data set. We thus conclude that the outcome of machine learning models, such as the one proposed here, requires analyzing MRI metrics as a whole to gain knowledge about the effect of alcohol on individuals.

Strong agreement existed among the regions included in the four informative patterns identified by Joi-GAM-Class. While inter-dependencies between the repeated training runs with varying parameter settings of Joi-GAM-Class could account for this finding, this explanation fails to explain the consistency between the informative patterns identified by Joi-GAM-Class and alternative implementations. A more likely explanation for this consistency is the significant impact of regular drinking on the regions identified by Joi-GAM-Class.

The brain regions identified by Joi-GAM-Class are relevant with respect to the Alcohol Use Disorder (AUD) literature. For example, the centrum semiovale, the most frequently appearing region across all patterns, was modestly smaller in the regular than in the minimal drinking group. This finding is consistent with *in vivo* neuroimaging^26^ and postmortem studies^33^ reporting smaller centrum semiovale volume in heavy alcohol drinking relative to healthy control adults. Smaller than normal white matter volumes could indicate a disruption in adolescent brain development given that white matter continues to grow throughout early adulthood^34–36^.

A number of studies report that the corpus callosum is sensitive to alcohol use disorder^26,37,38^. The corpus callosum integrates information and mediates complex behaviors^39^ and is larger and thicker in adolescents with higher intelligence^40,41^ and better problem solving abilities^42^. The cingulate cortex has been associated with selective attention^43^, conflict monitoring and decision making in controls^44^ and alcoholics^45,46^. The lateral ventricles are generally enlarged in heavy alcohol consuming adults and serve as a sensitive marker of alcoholic-level drinking^13,47,48^.

Joi-GAM-Class also identified regions with altered DTI metrics in the regular drinkers relative to the minimal drinking adolescents. Although low fractional anisotropy and high mean radial diffusivity are often reported in heavy drinking youth^15^, the current study reports that axial diffusivity of the fornix, fractional anisotropy of the anterior corona radiata and posterior thalamic radiation were high in the regular drinking group. These findings were also reported previously^4^, albeit at a statistically insignificant level. A recent longitudinal study of detoxified alcohol-dependent male adolescents found evidence of low white matter integrity in the body of the anterior corona radiata^15^. Microstructural compromise of the fornix, a major fiber bundle connecting limbic structures, has been reported in adult alcoholics^49^.

We complete the review of our morphological findings by noting that the importance of single-region metrics (*i*.*e*., its frequency of appearance in the training runs as specified in Table 2) was unrelated to its significance in discriminating the two cohorts, *i*.*e*., only half the scores were significantly different between groups. The importance of a single-region metric was also unrelated to its accuracy in classification, *i*.*e*., all single-regional metrics reported low matched-accuracies. These observations were further supported by repeating two-fold cross-validation of the sequential procedure with the classifier (without sparsity constraints) being trained on the 29 regional measurements. These 29 MRI metrics were identified by applying a two-tailed t-test to residual scores of the training dataset and retaining those with $p\le 0.01$ (*i*.*e*., the significance threshold that led to the highest classification accuracy). The resulting normalized-accuracy of the classifier based on these 29 metrics at 67.3% was significantly lower than that of Joi-GAM-Class. Thus, the type of machine learning applied here analyzed all potentially informative metrics as a whole^12^ to determine those regions impacted by regular alcohol use on the developing adolescent brain. In support of this statement, Joi-GAM-Class received lower accuracy scores than those listed in Table 2 for ‘Pattern 4’, the informative pattern of Joi-GAM-Class consisting of all eight regional scores. This pattern is the first known imaging marker with respect to the NCANDA cohort that predicts (*i*.*e*., classified with significant accuracy) individuals with regular drinking habits at baseline.

For readers interested in the technical aspects of our proposed machine learning approach, the remainder of the discussion focuses on differences in the implementations and their impact on accuracy scores. We first note, that No-GAM-Class, *i.e*., performing classification without the GAM model, failed the age-test and resulted in low accuracy scores, thereby supporting the need for properly modeling the effects of confounding factors. One way of modeling the effect is to perform the analysis on a subset of the data with the cohorts being carefully matched with respect to confounding factors. However, the sample size of a matched data set is often much smaller than the original dataset, thereby reducing statistical power. Alternatively, the effects of confounding factors can be removed via GAM.

When parameterizing a GAM independently from classification (*i*.*e*., sequential approaches), the residual effects of confounding factors can significantly effect the final classification, as observed here, since the sequential approaches (Seq-GAM-Class and Seq-GAM_*Rob*_-Class) failed the age-test. That the joint implementations Joi_*DTI*_-GAM-Class and Joi_*OPT*_-GAM-Class also failed the age test is a caution to check the output of regression-based approaches for the effects of confounding factors. The series of stringent statistical tests performed post hoc in this study identified those outputs that were not selected because of contributions of confounding factors. Based on those tests, the only informative patterns were determined by the joint implementations Joi_*STR*_-GAM-Class and Joi-GAM-Class.

When confining the joint analysis to just one modality, classification achieved higher accuracy when based on structural metrics (*i.e*., Joi_*STR*_-GAM-Class) than when based on DTI metrics (*i.e*., Joi_*DTI*_-GAM-Class). The higher accuracy scores of Joi-GAM-Class over the single-modality implementations (*i.e*., Joi_*STR*_-GAM-Class and Joi_*DTI*_-GAM-Class) further highlight the importance of analyzing multiple modalities together.

In conclusion, only the joint approaches Joi_*STR*_-GAM-Class and Joi-GAM-Class passed the age-test, showed significant normalized- and matched-accuracy scores, and succeeded in identifying informative patterns on a data set not ideally constructed for classification. Thus, our experiments support the hypothesis of this study.

## Methods

### Participants

At baseline^4^, NCANDA recruited 831 adolescents, of whom 28 were excluded for the current analysis due to brain abnormalities or missing MRI data. Of the remaining 803 youth, 671 (333 male and 338 female adolescents, ages 12 to 21 years) met the criteria for minimal (no-to-low) alcohol consumption^17^ and comprised the control group. The remaining 132 adolescents reported initiating moderate-to-heavy alcohol consumption: female participants consumed four or more drinks (beer, wine, or hard liquor) and male participants consumed five or more drinks (beer, wine, or hard liquor) on at least one occasion in their lifetime. Of these, 34 subjects met criteria for regular drinking (*i*.*e*., they drank a minimum of two alcoholic drinks at least once per week). The total data set on 705 youth (671 minimal and 34 regular drinkers) used in this study included demographic information and MRI scans acquired across the five NCANDA collection sites^17^, two of which used Siemens 3 T Tim Trio scanners (Siemens) and three of which used General Electric 3 T Discovery MR750 scanners (GE). Each participant was described by age, sex, self-reported ethnicity, socio-economic status (based on the highest education achieved by either parent)^50^, MRI scanner type (GE or Siemens), and supratentorial volume (determined from MR images) (see Table 3).

**Table 3:** Demographics of the NCANDA Samples (*N* = 705) and corresponding p-values between the regular drinkers and minimal alcohol exposed cohort. The two cohorts happen to be properly matched (p >  0.1) with respect to all demographic factors but age (in years) and socioeconomic status, whose p-values are marked in bold. The statistic of the supratentorial volume is listed in cm^3^.

		Minimal	Regular	p-value
		*N* = 671	*N* = 34	
Age	mean	15.7	19.5	**<0.0001**
	standard deviation	2.4	1.7	
Socioeconomic Status	mean	16.7	18	**0.0039**
	standard deviation	2.5	2.3	
Supratentorial Volume	mean	1248.7	1236.4	0.5737
	standard deviation	127.2	126.4	
Sex	Male	333	16	0.7701
	Female	338	18	
Ethnicity	*N*			0.7398
Caucasian		492	25	
African-American		86	6	
Asian		54	3	
Pacific-Islander		4	0	
Native-American		3	0	
Mixed		32	0	
Scanner	*N*			0.4032
GE		447	25	
Siemens		224	9	

The two cohorts were matched (*p* > 0.1) on ethnicity (multinomial Chi-Square test^51^), and sex, and MRI scanner type (binomial Chi-Square test^52^). Age, socio-economic status, and supratentorial volume (*i.e*., the only other confounding factors^3^) were compared using unpaired, two-tailed t-tests^53^. The two cohorts matched with respect to supratentorial volume but not age and socio-economic status. Most of the regular drinkers were older (18 or older) and had higher socio-economic status than the control group.

Brain imaging metrics used for each individual included 32 MRI derived structural volume scores extracted from the T1- and T2-weighted MRIs^3^, and 112 DTI derived microstructural scores^4^. All scores were provided as data releases (Demographic Score Release: NCANDA DATA 00010 V5, Structural Score Release: NCANDA DATA 00011, DTI Score Release: NCANDA DATA 00012 V2) by the software platform Scalable Informatics for Biomedical Imaging Studies (SIBIS; sibis.sri.com)^54^. The Section ‘Data Pre-processing’ of the supplement summarizes the pre-processing steps performed on these data as described by^3,4^.

### Implementations

All implementations used here were based on the sparse-logistic classification model^12^. This method is trained to accurately classify samples by minimizing an energy function that encodes the underlying classification task as finding informative patterns (of MRI metrics) of a certain size. No-GAM-Class directly trained the classifier on the 144 raw imaging metrics of each subject. Training of the sequential approaches Seq-GAM-Class and Seq-GAM_*Rob*_-Class consisted of first parameterizing a GAM for regressing out the effects of confounding factors (*i*.*e*., age and socio-economic status) before optimizing the classifier on the residual scores. The GAM used a linear model for capturing the relationship between the image metrics and socio-economic status and a quadratic model for capturing the relationship between the image metrics and age^3,4^. Seq-GAM-Class used the least square estimation and Seq-GAM_*Rob*_-Class used the robust regression (*i*.*e*., bisquare estimation, the default of *‘robustfit’* in Matlab2013b)^28^ to determine the optimal setting of GAM on the minimal drinkers of the training data set. The joint approaches (Joi_*STR*_-GAM-Class, Joi_*DTI*_-GAM-Class, Joi_*OPT*_-GAM-Class, and Joi-GAM-Class) removed the effects of confounding factors while concurrently optimizing classification accuracy by embedding the GAM model into the energy function of the classifier. While Joi_*OPT*_-GAM-Class reported the result with respect to minimizing the energy function, Joi_*STR*_-GAM-Class, Joi_*DTI*_-GAM-Class, and Joi-GAM-Class went one step further and extended the energy function so that it accounted for accuracy of the GAM in removing the effects of the confounding factors. Joi-GAM-Class (as well as Joi_*OPT*_-GAM-Class) considered all 144 imaging metrics, while Joi_*STR*_-GAM-Class was confined to the 32 macro-structural MRI metrics and Joi_*DTI*_-GAM-Class to the 112 micro-structural DTI metrics. The accuracy of each implementation was measured via 2-fold cross-validation described in further detail in the supplemental section on ‘Cross-Validation’.

For the technically inclined reader, the following subsections describe in detail the optimization algorithms used for training the sequential and joint implementations.

### Training of the Sequential Approaches

The training of a sequential approach consisted of two steps: (1) determine the optimal setting of the GAM with respect to the ‘control group’ (*i*.*e*., minimal drinking cohort) and (2) identify the pattern, *i*.*e*., the subset of residual imaging scores most informative for group separation. The pattern was identified by computing the ‘weights’ of a sparse, logistic regression classifier^12^ that resulted in the highest normalized-accuracy based on the training data.

To determine the optimal setting ${\alpha }_{i}\,:\,=({\alpha }_{i\mathrm{,0}},\ldots ,{\alpha }_{i\mathrm{,3}})$ of the GAM with respect to each image measurement type ‘*i*’, let ‘*age*_*s*_’ be that age and ‘*ses*_*s*_’ the socio-economic status of subject ‘*s*’. Then the GAM defined the relationship of the confounding factors to the corresponding image score *i*_*s*_ as

$${i}_{s} \sim {\alpha }_{i,0}+{\alpha }_{i,1}\cdot ag{e}_{s}+{\alpha }_{i,2}\cdot ag{e}_{s}^{2}+{\alpha }_{i,3}\cdot se{s}_{s}.$$

Assuming that the image scores were Gaussian distributed, then determining the optimal *α*_*i*_ was equivalent to maximizing a likelihood function parameterized by the mean of a Gaussian distribution. To define the likelihood function, we now introduce the mathematical notation summarized in Table S1 of the supplement. Specifically, the training data (*i.e*., one of the folds) consisted of two cohorts totaling *N* = 352 subjects with ${\mathbb{C}}$ representing the set of indices of the minimal drinkers. Each subject ‘*s*’ was described by the factor vector ${{\bf{d}}}_{s}\mathrm{=[1,}\,ag{e}_{s},ag{e}_{s}^{2},se{s}_{s}]$ (consisting of *N*_*D*_ = 3 subject specific demographic values) and up to ${N}_{F}=144$ image scores **i**_*s*_. Training the GAM with respect to the data of the non-drinking cohort was then equivalent to fitting a matrix Φ so that the factor vector of each control subject was a predictor of the corresponding image scores *i.e*., ${{\bf{i}}}_{s} \sim {\rm{\Phi }}\cdot {{\bf{d}}}_{s}^{{\rm{{\rm T}}}}$. Assuming that ${{\bf{i}}}_{s} \sim {\mathscr{N}}({\rm{\Phi }}\cdot {{\bf{d}}}_{s}^{{\rm{{\rm T}}}},\,{\sqrt[{N}_{D}]{{\sigma }_{s}}}^{2})$ was normally distributed with ${\sigma }_{s}^{2}\in {\mathbb{R}}$ and referring to ${\Vert \cdot \Vert }_{2}$ as the *l*_2_-norm, the optimal fitted matrix $\hat{{\rm{\Phi }}}$ was obtained by solving the following maximum likelihood problem

1 $$\begin{array}{lll}\hat{{\rm{\Phi }}} & := & \text{arg}\mathop{\max }\limits_{{\rm{\Phi }}}P(|D,\,{\rm{\Phi }})={\rm{\arg }}\mathop{{\rm{\max }}}\limits_{{\rm{\Phi }}}\prod _{s\in {\mathbb{C}}}P({{\bf{i}}}_{s}|{{\bf{d}}}_{s},{\rm{\Phi }},{\sigma }_{s})={\rm{\arg }}\mathop{{\rm{\max }}}\limits_{{\rm{\Phi }}}\prod _{s\in {\mathbb{C}}}\frac{1}{{\sigma }_{s}\sqrt[ND]{2\pi }}{e}^{-\frac{1}{2{\sigma }_{s}^{2}}{({{\bf{i}}}_{s}-{\rm{\Phi }}\cdot {{\bf{d}}}_{s}^{{\rm T}})}^{T}({{\bf{i}}}_{s}-{\rm{\Phi }}\cdot {{\bf{d}}}_{s}^{{\rm T}})}\\  & = & {\rm{\arg }}\,\mathop{\min }\limits_{{\rm{\Phi }}}G({\rm{\Phi }})\,{\rm{with}}\,G({\rm{\Phi }})\,:\,=\sum _{s\in {\mathbb{C}}}\frac{1}{{\sigma }_{s}^{2}}\parallel {\rm{\Phi }}\cdot {{\bf{d}}}_{s}^{{\rm{{\rm T}}}}-{{\bf{i}}}_{s}{\parallel }_{2}^{2},\end{array}$$

where *D* is the set of factor vectors and *I* is the corresponding set of image scores across all samples.

Interpreting $\mathrm{1/}{\sigma }_{s}^{2}$ as the ‘weight’ of each sample, the above minimization problem defined a robust regression of Φ that was solved via bi-square estimation. With respect to the ordinary GAM, ${\sigma }_{s}=\sigma$ was assumed to be uniform across all subjects so that computing $\hat{{\rm{\Phi }}}$ simplified to the least-square solution. Regardless of the specific computation of $\hat{{\rm{\Phi }}}$, the corresponding residual (desensitized) scores of all *N* subjects were determined via

2 $$R\,:=[{{\bf{r}}}_{1},{{\bf{r}}}_{2},\mathrm{..}.,{{\bf{r}}}_{N}]\,\,{\rm{with}}\,{{\bf{r}}}_{s}(\hat{{\rm{\Phi }}})\,:={{\bf{i}}}_{s}-\hat{{\rm{\Phi }}}\cdot {{\bf{d}}}_{s}^{{\rm{{\rm T}}}}.$$

Training of the sparse, logistic regression classifier consisted of minimizing a log probability with respect to the weights selecting the subset of informative residual image scores best separating both cohorts. In order to define the log probability, the association of each subjects ‘$s$’ to a cohort was encoded by label ${z}_{s}$. If the subject was a regular drinker then ${z}_{s}=1$, and ${z}_{s}=-\,1$ if it was a minimal exposed individual. $Z\,:\,=[{z}_{1},{z}_{2},\mathrm{..}.,{z}_{N}]$ was the vector of label assignments of all subjects in the training fold. The logistic function was defined as $\theta (a)\,:\,=\,\mathrm{log}\,\mathrm{(1}+\exp (\,-\,a))$, the weight vector ‘*ω*’ encoded the importance of each residual score of **r**_*s*_ in distinguishing the two cohorts, and $v\in {\mathbb{R}}$ was the ‘label offset’. Assuming that all samples were independently and identically distributed according to the binomial distribution

3 $$P({z}_{s}|{{\bf{r}}}_{s}(\hat{{\rm{\Phi }}}),\nu ,\omega )\,:\,=\frac{1}{1+{e}^{-{z}_{s}\cdot ({\omega }^{T}\cdot {{\bf{r}}}_{s}(\hat{{\rm{\Phi }}})+\nu )}}=\frac{1}{1+{e}^{-{z}_{s}\cdot ({\omega }^{T}\cdot ({{\bf{i}}}_{s}-\hat{{\rm{\Phi }}}\cdot {{\bf{d}}}_{s}^{{\rm T}})+\nu )}}=P({z}_{s}|{{\bf{i}}}_{s},{{\bf{d}}}_{s},\hat{U},\nu ,\omega ),$$

determining the optimal parameters was equivalent to minimizing the following logistic cost function

4 $$L(\hat{{\rm{\Phi }}},\nu ,\omega )\,:\,=-\,\mathrm{log}(\prod _{s=1}^{N}P({z}_{s}|{{\bf{r}}}_{s}(\hat{{\rm{\Phi }}}),\nu ,\omega ))=\sum _{s=1}^{N}\theta ({z}_{s}\cdot ({\omega }^{{\rm{{\rm T}}}}\cdot {{\bf{r}}}_{s}(\hat{{\rm{\Phi }}})+\nu ))$$

with respect to a sparse search space defined according to the *l*_0_-‘norm’ ${\Vert \cdot \Vert }_{0}$ and a predefined number *N*_*K*_ < *N*_*F*_ of non-zero elements, *i.e*., the sparsity constraint

5 $${{\mathbb{S}}}_{{N}_{K}}\,:\,=\{\omega :{\Vert \omega \Vert }_{0}\le {N}_{K}\}.$$

In other words, parameterizing the sparse, logistic classifier summarizes to determining the optimal parameters $\hat{\nu }$ and $\hat{\omega }$ for the following minimization problem

6 $$(\hat{\nu },\hat{\omega })\,:\,={\rm{\arg }}\,\mathop{\min }\limits_{\nu \in {\mathbb{R}},\omega \in {{\mathbb{S}}}_{{N}_{K}}}L(\hat{{\rm{\Phi }}},\nu ,\omega \mathrm{)}.$$

We determined its solution via penalty decomposition^12^. The image scores associated with non-zero entries in $\hat{\omega }$ then defined the group separating pattern.

### Training of Joint Methods

Alternative to the sequential approach, the training of the joint approach consisted of simultaneously determining the optimal values for the variables $\hat{{\rm{\Phi }}}$ of the GAM and $(\hat{\nu },\hat{\omega })$ of the sparse, logistic regression. Specifically, the joint approaches were parameterized by maximizing the following joint probability

7 $$P(Z,I|{\rm{\Phi }},v,\omega ,D)\,:\,=\prod _{s\mathrm{=1}}^{N}P({z}_{s},{{\bf{i}}}_{s}|{{\bf{d}}}_{s},{\rm{\Phi }},v,\omega ,\sigma )=\prod _{s\mathrm{=1}}^{N}P({z}_{s}|{{\bf{i}}}_{s}{\boldsymbol{,}}{{\bf{d}}}_{s},{\rm{\Phi }},v,\omega )\cdot P({{\bf{i}}}_{s}|{{\bf{d}}}_{s},{\rm{\Phi }},\sigma \mathrm{)}.$$

$P({z}_{s}|{{\bf{i}}}_{s},{{\bf{d}}}_{s},{\rm{\Phi }},\nu ,\omega )$ was defined according to Eq. (3) and $P({{\bf{i}}}_{s}|{{\bf{d}}}_{s},{\rm{\Phi }},\sigma )$ according to the normal distribution of Eq. (1). Computing the log of that joint probability resulted in

8 $$\mathrm{log}\,P(Z,I|{\rm{\Phi }},\nu ,\omega ,D)=-\,\sum _{s=1}^{N}\theta ({z}_{s}\cdot ({\omega }^{T}\cdot ({{\bf{i}}}_{s}-{\rm{\Phi }}\cdot {{\bf{d}}}_{s}^{T})+\nu ))-\,\frac{1}{2{\sigma }^{2}}\sum _{s=1}^{N}||{{\bf{i}}}_{s}-{\rm{\Phi }}\cdot {{\bf{d}}}_{s}^{T}{||}_{2}^{2}.$$

The previous section parameterized the GAM with respect to the minimal drinkers (controls) so that any significant deviation in the image scores of the second cohort could be directly related to the existing clinical literature. To comply with that model, we confined the second sum of Eq. (8) to the controls and model the ‘input’ of the regular drinkers in parameterizing the GAM through the uninformative, uniform distribution represented by the constant $c\in {\mathbb{R}}$. Thus, the log of the joint probability is redefined as

9 $$\mathrm{log}\,P(Z,I|{\rm{\Phi }},\nu ,\omega ,D)=-\,\sum _{s=1}^{N}\theta ({z}_{s}\cdot ({\omega }^{T}\cdot ({{\bf{i}}}_{s}-{\rm{\Phi }}\cdot {{\bf{d}}}_{s}^{T})+\nu ))-\frac{1}{2{\sigma }^{2}}\sum _{s\in {\mathbb{C}}}||{{\bf{i}}}_{s}-{\rm{\Phi }}\cdot {{\bf{d}}}_{s}^{T}|{|}_{2}^{2}+c,$$

and its minimization as

10 $$\begin{array}{lll}(\hat{{\rm{\Phi }}},\hat{\nu },\hat{\omega }) & := & {\rm{\arg }}\mathop{\min }\limits_{{\rm{\Phi }},\nu ,\omega ,\in {{\mathbb{S}}}_{{N}_{K}}}-\,\mathrm{log}\,P(Z,I|{\rm{\Phi }},\nu ,\omega ,D)\\  & = & {\rm{\arg }}\mathop{{\rm{\min }}}\limits_{{\rm{\Phi }},\nu ,\omega ,\in {{\mathbb{S}}}_{{N}_{K}}}\mathrm{(1}-\gamma )\cdot L({\rm{\Phi }},\nu ,\omega )+\gamma \cdot G({\rm{\Phi }}),\end{array}$$

where $\gamma \,:\,=\frac{1}{2{\sigma }^{2}+1}$ weighted the importance between the logistic cost function and the GAM, $\sigma$ was fixed beforehand via parameter exploration, *L*(·) was defined according to Eq. (4), and *G*(·) according to Eq. (1). Note, if *γ* = 0 then the above equation simplifies to Joi_*OPT*_-GAM-Class, the commonly used logistic regressor with the training of the GAM solely driven by group separation.

Given that finding the optimal solution of Eq. (10) was prone to a local minimum due to the non-convex energy function, the parameters Φ′ were initialized by the output of the GAM of Eq. (1). The local minimum for Equation (10) was then determined through an algorithm inspired by penalty decomposition^12^. Specifically, we introduced $\hat{\psi }\in {\mathbb{S}}={{\mathbb{R}}}^{{N}_{F}}$, the non-sparse approximation of the weights $\hat{\omega }$. Eq. (10) was then equivalent to

11 $$(\hat{{\rm{\Phi }}},\hat{\nu },\hat{\psi },\hat{\omega })={\rm{\arg }}\mathop{{\rm{\min }}}\limits_{{\rm{\Phi }},\nu ,\psi \in {\mathbb{S}},\omega \in {{\mathbb{S}}}_{{N}_{K}}}(1-\gamma )\cdot L({\rm{\Phi }},\nu ,\psi )+\gamma \cdot G({\rm{\Phi }})\,{\rm{s}}{\rm{.t}}.\,\omega -\psi =0.$$

Introducing the weighting parameter $\rho  > 0$, the solution of Eq. (11) was iteratively estimated by

12 $$({{\rm{\Phi }}}_{\rho },{\nu }_{\rho },{\psi }_{\rho },{\omega }_{\rho })\,:\,={\rm{\arg }}\mathop{{\rm{\min }}}\limits_{{\rm{\Phi }},\nu ,\psi ,\in {\mathbb{S}},\omega \in {{\mathbb{S}}}_{{N}_{K}}}\mathrm{(1}-\gamma )\cdot L({\rm{\Phi }},\nu ,\psi )+\gamma \cdot G({\rm{\Phi }})+\rho \cdot \parallel \omega -\psi {\parallel }_{2}^{2}.$$

**Algorithm 1:**
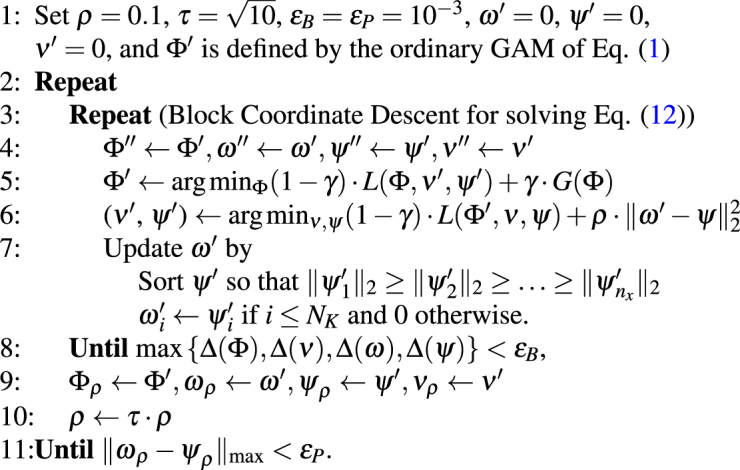
Jointly Parameterizing GAM and Classification.

As pointed out in Algorithm 1, the parameters of the logistic regression model were initialized as $\omega =0$, $\psi =0$, $\nu =0$^12^ and then, together with Φ, updated via block coordinate descent. If the parameters converged, $\rho$ was increased and the procedures was repeated until the maximum of the absolute difference between the elements of the sparse weights *ω*_*ρ*_ and the non-sparse weights *ψ*_*ρ*_ was below a fixed threshold ${\varepsilon }_{P}$^12^, *i*.*e*., let ${\Vert \cdot \Vert }_{{\rm{\max }}}$ denote the maximum element of a vector or matrix then

13 $$\parallel {\omega }_{\rho }-{\psi }_{\rho }{\parallel }_{{\rm{\max }}} < {\varepsilon }_{P}.$$

At each of these iterations, block coordinate descent improved the current estimate Φ′, ν′, *ω*′, and *ψ*′ of (Φ_*ρ*_, ν_*ρ*_, *ω*_*ρ*_, *ψ*_*ρ*_) by minimizing Eq. (12) fixing all variables but one and repeating this process until all variables converged. Keeping ν′, *ω*′, and *ψ*′ fixed, then the minimization problem of Eq. (12) simplified to

14 $${\rm{\Phi }}^{\prime} ={\rm{\arg }}\,\mathop{\min }\limits_{{\rm{\Phi }}}\mathrm{\ (1}-\gamma )\cdot L({\rm{\Phi }},\nu ^{\prime} ,\psi ^{\prime} )+\gamma \cdot G({\rm{\Phi }}\mathrm{)}.$$

Since the penalty function was smooth and convex, Eq. (14) was solved via gradient descent. Interpreting the above minimization problem as desensitizing the image scores from the influence of demographic factors, Φ parameterized the GAM specified by *G*(Φ) and was regularized by *L* (·, ν′, *ψ*′) to account for the noise in the image measurements **i**_*s*_.

Next, block coordinate descent updated ν′ and *ψ*′ by keeping Φ′ and *ω*′ fixed so that Eq. (12) simplified to

15 $$(\nu ^{\prime} ,\psi ^{\prime} )={\rm{\arg }}\mathop{\min }\limits_{\nu ,\psi ,\in {\mathbb{S}}}\mathrm{(1}-\gamma )\cdot L({\rm{\Phi }}^{\prime} ,\nu ,\psi )+\rho \cdot \parallel \omega ^{\prime} -\psi {\parallel }_{2}^{2}.$$

Again, gradient descent was employed to determine the minimum of that equation as the penalty function was smooth and convex. Finally, *ω*′ was updated by solving Eq. (12) with fixed Φ′, ν′, and *ψ*′, *i*.*e*., using the closed form solution to determine

16 $$\omega ^{\prime} \,:\,={\rm{\arg }}\mathop{{\rm{\min }}}\limits_{\omega \in {{\mathbb{S}}}_{{N}_{K}}}\parallel \omega -\psi ^{\prime} {\parallel }_{2}^{2}.$$

Following the suggestion of Zhang *et al*.^12^, block coordinate descent was repeated (*i*.*e*., Equations (14–16) until the relative changes of Φ′, ν′, *ω*′, and *ψ*′, between iterations were smaller than a fixed threshold ${\varepsilon }_{B}$, *i*.*e*.

17 $${\rm{\max }}\,\{{\rm{\Delta }}({\rm{\Phi }}),{\rm{\Delta }}(\nu ),{\rm{\Delta }}(\omega ),{\rm{\Delta }}(\psi )\} < {\varepsilon }_{B}.$$

with ${\rm{\Delta }}(a)=\frac{\parallel a^{\prime} -a^{\prime\prime} {\parallel }_{{\rm{\max }}}}{{\rm{\max }}\,\{\parallel \,a^{\prime} \,{\parallel }_{{\rm{\max }}}\mathrm{,1\}}}$. Once converged, Φ_*ρ*_, ν_*ρ*_, *ω*_*ρ*_, and *ψ*_*ρ*_ were updated according to Φ′, ν′, *ω*′, and *ψ′*, *ρ* was increased, and another block coordinate descent was initiated until *ω*_*ρ*_ and *ψ*_*ρ*_ converged according to Eq. (13), which was the case in all of our experiments. Additional comments about the joint optimization are provided by the supplement.

### Data availability

In compliance with NIH policy, the data release NCANDA DATA 00010 V5, NCANDA DATA 00011, and NCANDA DATA 00012 V2 that supports the finding of this study is released to the public according to the NCANDA Data Distribution agreement (see https://www.niaaa.nih.gov/research/major-initiatives/national-consortium-alcohol-and-neurodevelopment-adolescence for more detail).

### Code availability

Our Matlab implementation of the proposed algorithm (GAM-Sparsity Constraint Logistic Regression V1) is available via https://www.nitrc.org/projects/gam_sparityreg.

### Informed Consent

All procedures performed in this study were in accordance with the Declaration of Helsinki. All participants underwent informed consent processes at the visit with a research associate trained in human subject research protocols. Adult participants or the parents of minor participants provided written informed consent before participation in the study. Minor participants provided assent before participation. The Institutional Review Boards of each NCANDA site approved this study, and each site followed this procedure to obtain voluntary informed consent or assent, depending on the age of the participant.

## Electronic supplementary material

Supplemental Material and Methods
