# Supplementary material for: Alcohol use effects on adolescent brain development revealed by simultaneously removing confounding factors, identifying morphometric patterns, and classifying individuals
Source: Sci Rep. 2018 May 29;8:8297. doi: 10.1038/s41598-018-26627-7 (PMC5974423; doi:10.1038/s41598-018-26627-7)
Supplement: Supplementary file 1 — Supplemental Material and Methods [file 41598_2018_26627_MOESM1_ESM.pdf]

Alcohol use effect on adolescent brain development revealed by simultaneously removing confounding factors, identifying morphometric patterns, and classifying individuals

Sang Hyun Park[1,+], **Yong Zhang**[2,+], **Dongjin Kwon**[3,4], **Qingyu Zhao**[3], **Natalie M. Zahr**[3,4], **Adolf Pfefferbaum**[3,4], **Edith V. Sullivan**[3], **and Kilian M. Pohl**[4,*]

[1]Department of Robotics Engineering, Daegu Gyeongbuk Institute of Science and Technology, South Korea
[2]Colin Artificial Intelligence Lab, Richmond, BC Canada
[3]Department of Psychiatry & Behavioral Sciences, Stanford University, Stanford, CA 94305 USA
[4]Center for Health Sciences, SRI International, Menlo Park, CA 94025, USA
[*]Corresponding author. E-mail: kilian.pohl@sri.com
[+]These authors contributed equally to this work

Supplemental Material and Methods

Data-Pre-Processing

According to Pfefferbaum et al.[1], the macro-structural scores were the result of processing structural MRIs via a pipeline that included skull stripping based on majority voting[2] across multiple maps generated by publicly available software packages and a label map based on the SRI24 atlas[3]. This yielded intracranial volume, supratentorial volume, pons, corpus callosum, subcortical white matter (including the centrum semiovale), and lateral ventricular volume. Furthermore, FreeSurfer[4] was applied to the skull-stripped MRIs to create bilateral surface area, volume, and thickness of frontal, temporal, parietal, occipital, cingulate cortices derived from the Desikan-Killiany atlas[5].

According to Pohl et al.[6], the micro-structural scores were generated by excluding bad single shots from DTI scans, correcting those scans for echo-planar distortion[7], and performing motion correction. All DTI scans were skull-stripped and applied to CAMINO's linear single tensor model approach[8] to infer fractional anisotropy, mean diffusivity, axial diffusivity, and radial diffusivity maps. The four maps were non-rigidly aligned to the SRI24 atlas[3] and corrected for scanner differences based on human phantom data[6]. The fractional anisotropy-corrected maps of all subjects were transformed into fractional anisotropy skeletons via Tract-Based Spatial Statistics[9] and the template of the fractional anisotropy skeleton was mapped to generate mean diffusivity, axial diffusivity, and radial diffusivity skeletons. Finally, the pipeline computed 112 DTI scores by reducing each skeleton to the mean values of 28 regions, which were defined according to the Johns Hopkins University atlas and its mask[10] aligned to the SRI24 atlas[6].

Cross-Validation

The accuracy of each implementation was measured via cross-validation. Cross-validation is a popular approach in the machine learning community as it minimizes the risk of reporting overly optimistic accuracy scores by repeatedly training and testing an implementation on separate subsets of the data. Specifically, the data set was divided into two non-overlapping subsets so that each subset preserved most of the characteristics of the complete data set (*e.g.*, for each subset, the ratio of samples between the two cohorts was consistent and the two cohorts were matched with respect to ethnicity, sex, scanner type, and supratentorial volume). For both subsets, the cohorts were not matched with respect to age ($p < 0.0001$). With respect to socioeconomic status[11], one subset matched ($p = 0.138$) and one did not (p = 0.013). Due to the relatively large p-value of socioeconomic status ($p = 0.0036$) and the small number of regular drinkers ($N = 34$) of the entire data set, having to match both subsets with respect to socioeconomic status would have required omitting samples from the study, which would have compromised the integrity of this analysis.

Each implementation was then trained on the first subset using a variety of algorithmic parameter settings. Specifically, the search space for the sparsity setting 'N_K' of the logistic classifier was bounded by the smallest pattern (consisting of more than one element) and half of the imaging scores, which for Joi$_{STR}$-GAM-Class was $N_K \in \{2,4,\ldots,16\}$, for Joi$_{DTI}$-GAM-Class was $N_K \in \{2,4,\ldots,56\}$, and for all other implementation was $N_K \in \{2,4,\ldots,72\}$. In addition, the robust regression of Seq-GAM$_{Rob}$-Class required setting the optimal 'scaling' parameter, for which the search range was $\{0,0.5,\ldots,6\}$. Note, the classification accuracy of Seq-GAM$_{Rob}$-Class on the training data varied by almost 5% depending on the specific setting of that parameter. Finally, the joint implementations (*i.e.*, Joi$_{STR}$-GAM-Class, Joi$_{DTI}$-GAM-Class, and Joi-GAM-Class) weighted the importance of the GAM model over the logistic classifier through the weight 'γ', which varied between $\{0.1,0.2,\ldots,0.9\}$ with $\gamma = 0.1$ focusing mostly on improving classification accuracy and with $\gamma = 0.9$ aiming to determine the optimal GAM model.

For each implementation and parameter setting, the training determined the optimal values for the GAM variables $\alpha_{i,0},\ldots,\alpha_{i,3}$ for each image score 'i' and selected the corresponding residual image scores (*i.e.*, patterns) that lead to the highest normalized-accuracy of the classifier on the training data. Computing the normalized-accuracy required first recording the accuracy of the classifier in correctly labeling subjects of the minimal alcohol exposed cohort and the accuracy of correctly labeling the regular drinkers, and then computing the average across the two resulting (cohort-specific) accuracy scores. For each implementation, the classifiers (and corresponding pattern) across all training runs (*i.e.*, setting) were then combined into a single ensemble of classifiers[12], which computed the weighted average across the decisions of all classifier with the weight of a classifier being defined by its training accuracy.

To 'test' the ensemble of classifiers of each implementation, it was applied to the second subset and the labeling decisions were recorded with respect to assigning a sample to the minimal alcohol exposed cohort or regular drinking cohort. The process of training and testing was repeated a second time using the second subset for training and the first subset for testing. The testing accuracy of each implementation was then summarized by computing a number of variables (*i.e.*, sensitivity, specificity, Area Under the receiver operating characteristic Curve (AUC), normalized-accuracy, matched-accuracy, age-test, and socioeconomic status test) with respect to the cohort assignment of the testing data. To compute the matched-accuracy, 34 minimal alcohol exposed adolescents were matched to the 34 regular drinking subjects with respect to all confounding factors (age: p=0.12; socioeconomic status: $p = 0.2$; supratentorial volume: $p = 0.61$; sex: $p = 1.0$; ethnicity: $p = 1.0$; scanner: $p = 1.0$). Then the normalized-accuracy was computed with the respect to the matched-set. Normalized-accuracy on the entire data set and

Table S1: Variables used in the mathematical models of the sequential and joint approach

N	the number of all subjects
N_F	the number of measurements
N_D	the number of demographic scores
s	a subject
$\mathbf{i}_s$	image scores of s
I	image scores of all subjects
$\mathbf{d}_s$	confounding scores of s
D	confounding scores of all subjects
$\mathbf{r}_s$	residual scores of s
R	residual scores of all subjects
z_s	label of s
Z	group labels of all subjects
Φ	parameters of GAM
σ_s	subject specific covariance of residual
σ	uniform covariance of residual across all subjects
ω	sparsity constrained weight vector of the logistic regression function
N_K	the upper bound on the number of non-zero elements in ω according to the sparsity constrain
$\mathbb{C}$	Set of indices of the control subjects
$\mathbb{S}_{N_K}$	sparse search space
$\mathbb{S}$	non-sparse search space
ν	label offset for logistic function
ψ	unconstrained weight vector for optimizing logistic function
ρ	parameter for penalty decomposition optimization
γ	weight between GAM and logistic function in joint model
c	constant representing the uninformative, uniform distribution of regular drinkers

matched-accuracy were labeled significant if the two-tailed Fisher's exact test[13] applied to classifications of the corresponding test data returned p <0.002.

To compute the age-test, the minimal alcohol exposed adolescents of the NCANDA data set were divided into an older (*i.e.*, above the age of 15.4 years) and younger (*i.e.*, below the age of 15.5 years) cohort, so that the cohorts were almost equal in size (older cohort: N=335; younger cohort: N=336) and matched with respect to all confounding factors but age (supratentorial volume : p= 0.4410, socioeconomic status: p=0.1277, sex: p=0.2026, race: p=0.1685, scanner: p=0.1334). The two-tailed Fisher's exact test was then applied to the earlier recorded labelings in order to see if that labeling was significantly better than chance in correctly assigning minimal alcohol exposed individuals to one of those two cohorts. Implementations passed the age-test if their $p > 0.01$, *i.e.*, the effect of age was magnitudes smaller than the effect of regular drinking for those implementations that reported significant normalized-accuracy and significant matched-accuracy.

Similar to the age-test, an implementation passed the socioeconomic status test if the two-tailed Fisher's exact test returned $p > 0.01$ with respect to the classification results correctly assigning minimal alcohol exposed individuals to the cohort with higher socioeconomic status (≥ 17) or lower socioeconomic status (< 17). These two cohorts, however, were only matched with respect to age (p=0.26), sex (p=0.18), and scanner (p=0.24) as socioeconomic status was highly correlated with ethnicity and supratentorial volume in minimal alcohol exposed individuals ($p < 0.001$ according to Pearson's correlation).

For each implementation, we also recorded the frequency of patterns appearing across all training runs. The frequency of a pattern of size 'N_K' was defined by the number of times it appeared as part of a pattern selected by a training run divided by the number of training runs that searched for patterns of at least size 'N_K'. This computation thus account for larger patterns not

Table S2: Acronyms

MRI	Magnetic resonance imaging
GAM	Generalized additive model
NCANDA	National Consortium on Alcohol and NeuroDevelopment in Adolescence
AUD	Alcohol use disorder
DTI	Diffusion tensor imaging
HIV	Human immunodeficiency virus
AUC	Area under the receiver operating characteristic curve
GE	3T General Electric Discovery MR750 scanner
Siemens	3T Siemens Tim Trio scanner
age-test	Test if classifier is impacted by the confounding factor age
No-GAM-Class	Sparsity constrained classification on the raw scores
Seq-GAM-Class	Sequential approach with ordinary GAM and sparsity-constrained classification
Seq-GAM$_{Rob}$-Class	Sequential approach with robust GAM and sparsity-constrained classification
Joi$_{STR}$-GAM-Class	Joint model confined to the structural measurements.
Joi$_{DTI}$-GAM-Class	Joint model confined to the DTI measurements.
Joi$_{OPT}$-GAM-Class	Joint model only optimizing for the group separation
Joi-GAM-Class	Joint model optimizing for regressing out confounding factors and the group separation

being able to be part of patterns selected by training runs searching for smaller ones. Patterns of an implementation were then labeled as highly informative for separating the two cohorts if their frequency was higher than 50%, *i.e.*, they appeared in a majority of patterns (of the same size or larger) selected by training runs.

Notes on Training of Sequential and Joint Methods

The computational time associated with convergence (*i.e.*, the training of the Matlab implementation of Joi-GAM-Class) was up to 5 minutes on a single core PC. One way of speeding up the training of the method is to replace Penalty Decomposition with a more commonly used sparse solver[14] that determined a sparse solution by relaxing the l_0-'norm' with the l_1-norm $\|\cdot\|_1$. The corresponding joint implementation again outperformed the corresponding sequential one (*i.e.*, sparse logistic classification based on the l_1-norm). However, this implementation was significantly less accurate than Joi-GAM-Class so that the results were omitted from Table 1 for clarity.

Once the joint method converged, one can readily show that applying the resulting optimal parameter setting ($\widehat{\Phi}, \hat{v}, \hat{\omega}$) to the joint or sequential approach results in the same classification. In other words, measuring the testing accuracy of the joint approach can be performed by first regressing out the effect of confounding factors from the raw imaging scores before applying a logistic classifier solely to the residual image scores, *i.e.*, the classifiers decision is done without knowing the confounding factors.

References

1. Pfefferbaum, D. *et al.* Adolescent development of cortical and white matter structure in the NCANDA sample: Role of sex, ethnicity, puberty, and alcohol drinking. *Cerebral Cortex* **26**, 4101–4121 (2016).
2. Rohlfing, T., Russakoff, D. B. & Maurer Jr, C. R. Performance-based classifier combination in atlas-based image segmentation using expectation-maximization parameter estimation. *IEEE Transactions on Medical Imaging* **23**, 983–994 (2004).
3. Rohlfing, T., Zahr, N. M., Sullivan, E. V. & Pfefferbaum, A. The SRI24 multi-channel atlas of normal adult human brain structure. *Human Brain Mapping* **31**, 798–819 (2010).
4. Dale, A. M., Fischl, B. & Sereno, M. I. Cortical surface-based analysis. I. Segmentation and surface reconstruction. *NeuroImage* **9**, 179–194 (1999).

5. Desikan, R. S. *et al.* An automated labeling system for subdividing the human cerebral cortex on MRI scans into gyral based regions of interest. *NeuroImage* **31**, 968–980 (2006).

6. Pohl, K. M. *et al.* Harmonizing DTI measurements across scanners to examine the development of white matter microstructure in 803 adolescents of the NCANDA study. *NeuroImage* **130**, 194–213 (2016).

7. Holland, D., Kuperman, J. M. & Dale, A. M. Efficient correction of inhomogeneous static magnetic field-induced distortion in Echo Planar Imaging. *NeuroImage* **50**, 175–183 (2010).

8. Cook, P. A. *et al.* Camino: Open-source diffusion-MRI reconstruction and processing . In *14th Scientific Meeting of the International Society for Magnetic Resonance in Medicine*, 2759 (2016).

9. Smith, S. M. *et al.* Tract-based spatial statistics: voxelwise analysis of multi-subject diffusion data. *NeuroImage* **31**, 1487–1505 (2006).

10. Mori, S., Wakana, S., Nagae-Poetscher, L. M. & Van Zijl, P. M. C. *MRI atlas of human white matter* (Elsevier, 2005).

11. Brown, S. A. *et al.* The National Consortium on Alcohol and NeuroDevelopment in Adolescence (NCANDA): A multi-site-study of adolescent development and substance use. *Journal of Studies on Alcohol and Drugs* **76**, 895–908 (2015).

12. Rokach, L. Ensemble-based classifiers. *Artificial Intelligence Review* **33**, 1–39 (2010).

13. Fisher, R. A. On the interpretation of χ^2 from contingency tables, and the calculation of P. *Journal of the Royal Statistical Society* **85**, 87–94 (1922).

14. Liu, J., Ji, S. & Ye, J. *SLEP: Sparse learning with efficient projections* (2011).
